# Design of an amphiphilic hyperbranched core/shell-type polymeric nanocarrier platform for drug delivery

**DOI:** 10.3906/kim-1910-35

**Published:** 2020-04-01

**Authors:** Ayça BAL ÖZTÜRK, Nesrin OĞUZ, Hande TEKARSLAN ŞAHİN, Serkan EMİK, Emine ALARÇİN

**Affiliations:** 1 Department of Analytical Chemistry, Faculty of Pharmacy, İstinye University, İstanbul Turkey; 2 Department of Stem Cell and Tissue Engineering, Institute of Health Sciences, İstinye University, İstanbul Turkey; 3 Department of Chemical Engineering, Faculty of Engineering, İstanbul University-Cerrahpaşa, İstanbul Turkey; 4 Beykoz Institute of Life Sciences and Biotechnology, Bezmiâlem Vakıf University, İstanbul Turkey; 5 Department of Pharmaceutical Technology, Faculty of Pharmacy, Marmara University, İstanbul Turkey

**Keywords:** Hyperbranched polymer, poly(aminoester), nanoparticle, drug delivery, 5−fluorouracil

## Abstract

An amphiphilic core/shell-type polymer-based drug carrier system (HPAE- PCL-b -MPEG), composed of hyperbranched poly(aminoester)-based polymer (HPAE) as the core building block and poly(ethylene glycol)-b - poly(ε-caprolactone) diblock polymers (MPEG-b -PCL) as the shell building block, was designed. The synthesized polymers were characterized with FTIR, 1 H NMR, 13 C NMR, and GPC analysis. Monodisperse HPAE-PCL-b - MPEG nanoparticles with dimensions of < 200 nm and polydispersity index of < 0.5 were prepared by nanoprecipitation method and characterized with SEM, particle size, and zeta potential analysis. 5-Fluorouracil was encapsulated within HPAE-PCL-b -MPEG nanoparticles. In vitro drug release profiles and cytotoxicity of blank and 5-fluorouracil-loaded nanoparticles were examined against the human colon cancer HCT116 cell line. All results suggest that HPAE-PCL-b - MPEG nanoparticles offer an alternative and effective drug nanocarrier system for drug delivery applications.

## 1. Introduction

The poor solubility and short lifetime of pharmaceutical drugs restrict their development and clinical application due to complicating factors associated with their manufacturing, poor absorption, stability, and bioavailability [1–3]. To overcome these challenges, nanoparticles can provide a promising and alternative strategy for the encapsulation of various molecules with improved efficacy and reduced side effects. There are various design and functionalization strategies for engineering drug-loaded nanoparticle formulations, such as nanocrystals, nanoemulsions, micelles, and polymersomes [4,5].

Among the different nanostructures employed in drug formulations, dendritic nanoparticles have recently gained much attention as a versatile platform due to their unique characteristics such as high solubility, low viscosity, superior stability, three-dimensional highly branched topology, and multifunctionality [6–9]. In addition, with the advantage of their great number of branches, they can encapsulate and deliver numerous drug molecules with higher encapsulation efficiency compared to linear polymeric micelles [10]. Dendritic polymers are divided into two major classes, namely dendrimers and hyperbranched polymers [5,7,8]. Though dendrimers are perfectly branched architectures with a degree of branching of 100%, they are not applicable in the pharmaceutical industry due to their costly and time-consuming synthesis. Hyperbranched polymers have random branch-on-branch topology and a figuration similar to that of dendrimers. Moreover, hyperbranched polymers are able to overcome the aforementioned shortcomings of dendrimers with a straightforward, applicable, and cost-effective synthesis strategy [5,7,11]. In addition, as drug carriers, hyperbranched polymers can offer their interior or terminal functional groups to covalently conjugate drug molecules or encapsulate them depending on the core-shell structure. Therefore, hyperbranched polymers emerge as good alternative nanocarriers to dendrimers [8].

PEGylation, one of the most important modification methods in therapeutic strategies, provides key advantages including reduction of immunogenicity and cytotoxicity, prolongation of blood circulation time, and improvement of solubility of both the drug and carrier systems. PCL, a semicrystalline and biodegradable polymer, was successfully used in medical devices and tissue engineering [12]. Copolymers of these polymers are biodegradable and biocompatible and have apparent hydrophilic and hydrophobic fragments generating core and shell building blocks due to their high solubility in aqueous solutions [12].

In the present study, we have designed an amphiphilic core-shell multifunctional polymeric nanocarrier (HPAE-PCL-b-MPEG) by grafting poly(ethylene glycol)-b-poly(ε-caprolactone) diblock polymers (MPEG-b- PCL) on the hyperbranched poly(aminoester)-based polymer (HPAE) backbone to evaluate their effectiveness for drug delivery applications. For this purpose, HPAE-PCL-b-MPEG polymer was synthesized as a result of a series of reactions and characterized. Then HPAE-PCL-b-MPEG nanoparticles were prepared by nanoprecipitation method. 5-Fluorouracil (5Fu) was used as a model drug and encapsulated within the nanoparticles. In vitro drug release profiles and cytotoxicity of these nanoparticles were examined against the human colon cancer HCT116 cell line. As a result, HPAE-PCL-b-MPEG nanoparticles were found to be an alternative and effective drug nanocarrier system for drug delivery applications.

## 2. Materials and methods

### 2.1. Materials

1,6-Hexanediamine, methyl acrylate, 5-fluorouracil (5Fu), poly(ethylene glycol) methyl ether (MPEG, Mw ~2000 Da), maleic anhydride (MA), ε-caprolactone (CL), N-(3-dimethylaminopropyl)-N’-ethylcarbodiimide hydrochloride (EDAC), stannous octoate (SnOct), and 4-(N,N-dimethylamino)pyridine (DMAP) were obtained from Aldrich Chemical Corporation. Dimethyl sulfoxide (DMSO), N,N-dimethylformamide (DMF), hexane, dichloromethane (DCM), methanol, and aluminum chloride were obtained from Merck. Ethoxylated trimethylolpropane (Et-TMP, Mn ~170) was provided as a gift by Perstorp Polyols AB, Sweden.

### 2.2. Synthesis of hyperbranched poly(aminoester) (HPAE)

The synthesis of HPAE was performed via the two-step procedure described by Kim et al. [13]. In the first step, 1,6-hexanediamine (20 mmol, 2.324 g) and methyl acrylate (1.5 mol, 129.14 g) were mixed with 150 mL of methanol in a round-bottomed glass reactor. The reaction mixture was stirred at room temperature for 3 days. Thereafter, methanol and the excess methyl acrylate were eliminated under reduced pressure and a transparent oily product was obtained (hexanediamine-tetraester). In the second step, the product (4.75 mmol, 2.18 g) was reacted with Et-TMP (4.75 mmol, 1.3 mL) via a bulk polycondensation reaction. Aluminum chloride (0.1 mol/L) was used as a catalyzer. The reaction mixture was stirred as the temperature was increased from 120 to 180 °C at a heating rate of 10 °C/h. After proceeding with the reaction for 7 h with vigorous stirring, DMF was added to dissolve the reaction product, followed by centrifugation to remove the AlCl3 and finally drying under vacuum. A honey-colored sticky solid product, HPAE, was obtained.

### 2.3. Synthesis of MPEG-b-PCL and MPEG-b-PCL-COOH

MPEG-b-PCL was synthesized in accordance with the literature by ring-opening polymerization of ε-caprolactone (CL) on poly(ethylene glycol) methyl ether (MPEG) chains in the presence of SnOct as a catalyst [14]. The product obtained was dissolved in DCM, collected by precipitation into cold methanol, and dried at room temperature under vacuum. A white-colored powder, MPEG-b-PCL, was obtained.

MPEG-b-PCL (1.11 mmol, 3 g), maleic anhydride (MA) (3.33 mmol, 0.33 g), and 4-(N, N-dimethylamino) pyridine (DMAP) (5.6 mg) were dissolved in 50 mL of DCM. The reaction mixture was stirred at 60 °C for 6 h under an inert gas atmosphere, precipitated into cold hexane, and finally dried at room temperature under vacuum. A white-colored powder, carboxylic acid-ended MPEG-b-PCL (MPEG-b-PCL-COOH), was obtained.

### 2.4. Synthesis of HPAE-PCL-b-MPEG

The solution of MPEG-b-PCL-COOH (0.9 mmol, 3 g) in 15 mL of DMSO was treated with EDAC (0.18 g) at room temperature under inert gas atmosphere for 1 h. Next, HPAE solution in DMSO (200 mg/mL) was added. After allowing the reaction to proceed for 24 h at room temperature, DMAP (0.129 g) was added and stirring was continued overnight at room temperature. After the dilution of the reaction mixture with deionized water, the mixture was placed in a dialysis membrane (MWCO = 12,000 Da, Sigma Co.) and dialyzed against deionized water for 3 days and finally lyophilized.

### 2.5. Preparation of HPAE-PCL-b-MPEG and HPAE-PCL-b-MPEG/5Fu nanoparticles

HPAE-PCL-b-MPEG nanoparticles were prepared in accordance with a rapid nanoprecipitation technique [15]. HPAE-PCL-b-MPEG (10 mg) was completely dissolved in 1 mL of DMF and added dropwise to 9 mL of deionized water under magnetic stirring (1000 rpm). After proceeding with magnetic stirring for 3 h, the solution was transferred to a dialysis tube (Spectrapore, MWCO 1000) and dialyzed against deionized water by replacing the medium every 15 min for 3 h to remove the organic solvent in a short time. 5Fu-loaded nanoparticles (HPAE-PCL-b-MPEG/5Fu) were prepared in accordance with the same procedure by adding the 5Fu to the polymer solution and then analyzed or lyophilized for 24 h before further analyses.

### 2.6. Characterization methods

The Fourier transform infrared (FTIR) spectra of the synthesized polymers in KBr pellets (sample/KBr = 1/200) were taken using a Digilab Excalibur-FTS 3000 MX model FT-IR spectrometer (USA). 1 H NMR and 13 C NMR spectra of the synthesized polymers were taken by dissolving samples in CDCl3 and DMF using the NMR Varian UNITY INOVA spectrometer (USA) operating at 500 MHz. The molecular weight of MPEG-b- PCL was evaluated by using GPC analysis (Malvern - OmniSEC). DMF was used as an eluent. The average sizes and size distributions of blank and drug-loaded polymeric nanoparticles were evaluated by dynamic laser scattering (DLS) using a Nanosizer (NanoZS Malvern Instruments, UK). SEM images were taken using a scanning electron microscope (Quanta FEG 450 SEM, USA) to understand the morphological characteristics of the prepared nanoparticles.

### 2.7. Drug loading and in vitro release studies

To find the optimal encapsulation conditions, different 5Fu loading experiments (polymer/drug weight ratio: 1/1, 1/0.5, 1/0.25, 1/0.1, and 1/0.05) were conducted. The freeze-dried HPAE-MPEG-b-PCL/5Fu nanoparticles were disturbed by ethanol and the concentration of encapsulated 5Fu in nanoparticles was recorded using a UVVis spectrophotometer (T80+ UV-VIS Double Beam, PG Instruments, UK) at 266 nm. Drug loading content (DLC) and drug loading efficiency (DLE) were calculated from Eq. (eq1) and Eq. (eq2) [16]:

(1)Drug loading content (DLC) (mg/g) =Amount of 5Fu in nanoparticlesAmount of nanoparticles

(2)Drug loading efficiency (DLE)(w/w%) =Amount of 5Fu in nanoparticles5Fu initially addedx100

In vitro drug release studies were performed using a dialysis technique [17]. The lyophilized HPAE-PCL-b- MPEG/5Fu nanoparticles (~3 mg) were dispersed in 5 mL of phosphate buffer solution (PBS, pH 7.4) and transferred into a dialysis tube (Spectrapore, MWCO 1000 Da) immersed in glass vials containing 50 mL of PBS. At predetermined time intervals, 1 mL of sample solution was removed from the release media and measured with a spectrophotometer at 266 nm. 5Fu release was calculated according to the calibration curve. The solution taken as a sample was replaced with fresh PBS in the release media.

### 2.8. In vitro cytotoxicity assay

Human colon cancer cells (HCT116) were grown in DMEM supplemented with 10% (v/v) fetal bovine serum (FBS) and 1% antibiotic-antimycotic solution (Wisent 450-115-EL). The cells were seeded into 96-well plates in triplicate at a concentration of 2 ×104 cells/well. After seeding, the plates were incubated at 37 °C with 5% CO2 for 24 h before the MTT assay. The culture media were removed and the cells were treated with free 5Fu, HPAE-PCL-b-MPEG, and HPAE-PCL-b-MPEG/5Fu nanoparticles at different concentrations (0.25, 0.50, 1, and 2 mg/mL) with fresh medium. After 24 h and 48 h of incubation, the wells were washed with PBS three times. Then 50 μL of 2 mg/mL MTT solution in PBS with 200 μL of fresh medium was added to each well and wells were incubated for 3 h at 37 °C. The medium was then removed and 200 μL of DMSO was added to dissolve the formazan crystals. The optical density of the plate was measured at 570 nm using a plate reader (Thermo Scientific Multiskan Ex, USA). Cell viability was calculated according to Eq. (eq3). Experiments were performed in triplicate.

(3)Cell viability (%) =absorbance of sampleabsorbance of controlx100

## 3. Results and discussion

### 3.1. Preparation and characterization of HPAE-PCL-b-MPEG

**Figure 1:**
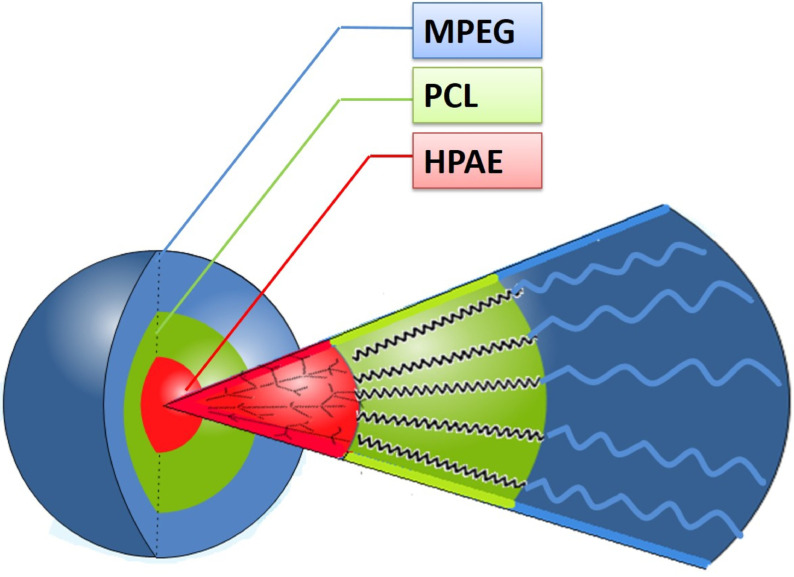
Building blocks and schematic representation of the formation of polymeric nanocarrier.

An amphiphilic hyperbranched core/shell-type polymeric nanocarrier (HPAE-PCL-b-MPEG) composed of a hyperbranched poly(aminoester)-based polymer (HPAE) as core building block and poly(ethylene glycol)-bpoly( ε-caprolactone) diblock polymers (MPEG-b-PCL) as shell building block was designed. A schematic representation of the amphiphilic hyperbranched core/shell-type polymeric nanocarrier (HPAE-PCL-b-MPEG) is illustrated in Figure 1.

**Figure 2:**
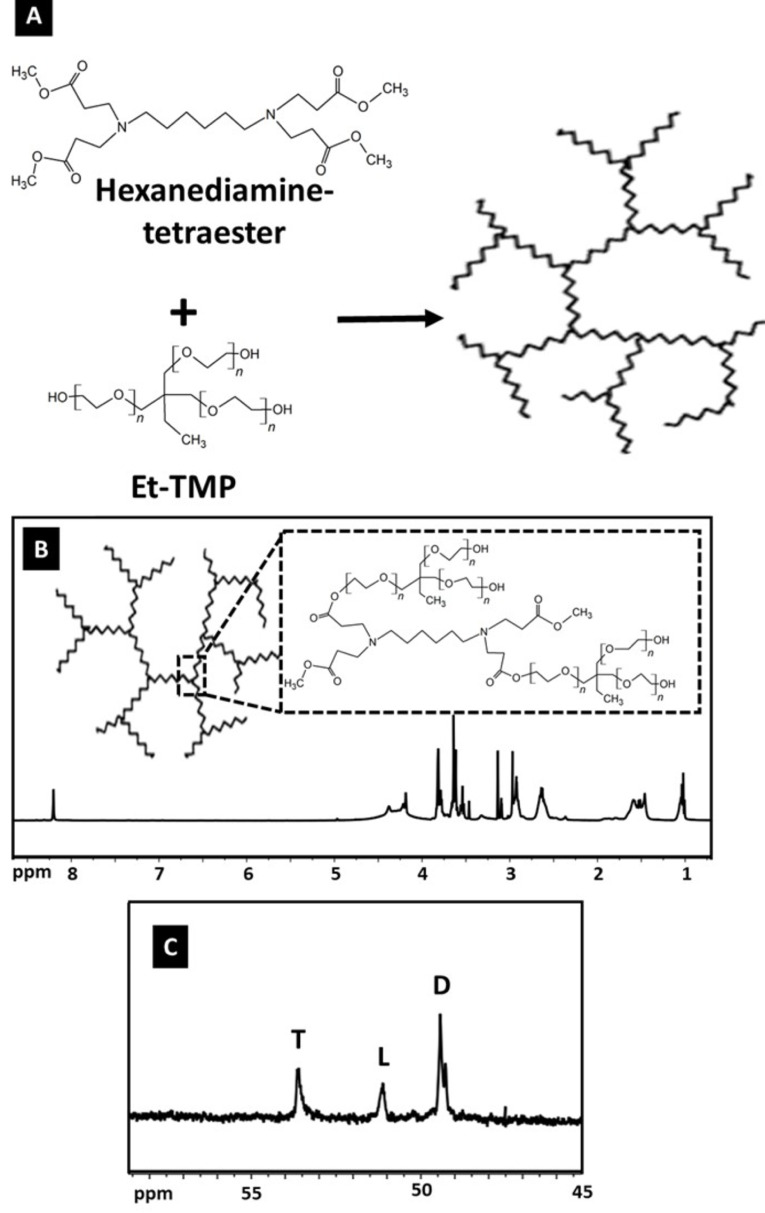
(A) The synthesis process of HPAE, (B) 1 H NMR of HPAE, and (C) internal structure of HPAE, 13 C NMR.

A two-step procedure was used to prepare the hyperbranched core molecule, HPAE: Michael addition and then polycondensation reaction (Figure 2A). HPAE was characterized by FTIR, 1 H NMR, and 13 C NMR. The basic chemical structure of HPAE was confirmed using 1 H and 13 C NMR (Figures 2B and 2C). It can be observed from the spectrum of HPAE that the characteristic peaks at 1.02 and 1.46 ppm were assigned to the methyl and methylene protons of –CH2 CH3 in the Et-TMP units. In addition, peaks at 2.64, 3.54, and 3.79 ppm were attributed to –CH2 COO–, –COOCH3 , and –CH2 CH2 COO–, respectively (Figure 2B). The degree of branching (DB) is used as a structural parameter to define and better understand the branched features of hyperbranched polymers [18]. The DB value of hyperbranched polymers is commonly determined by using integral intensities of the definite signals in NMR spectra. According to the Hawker and Fréchet definition, the DB value is equal to 1 for perfect dendrimers, whereas hyperbranched polymers usually display DB values between 0 and 1 [19]. The DB of HPAE was calculated according to Eq. (4) as follows:

DB = (D + T) / (D + L + T) (4)

Here, L is the number of linear groups, T is the number of terminal groups, and D is the number of dendritic groups. The peaks occurring at 49.43, 51.13, and 53.61 ppm were attributed to the signals of dendritic, linear, and terminal groups, respectively. In this study, the DB values of the HPAE polymer calculated on the basis of these three peaks (Figure 2C) using the definition of Hawker and Fréchet was found to be 0.84, which confirms the formation of highly branched products.

**Figure 3:**
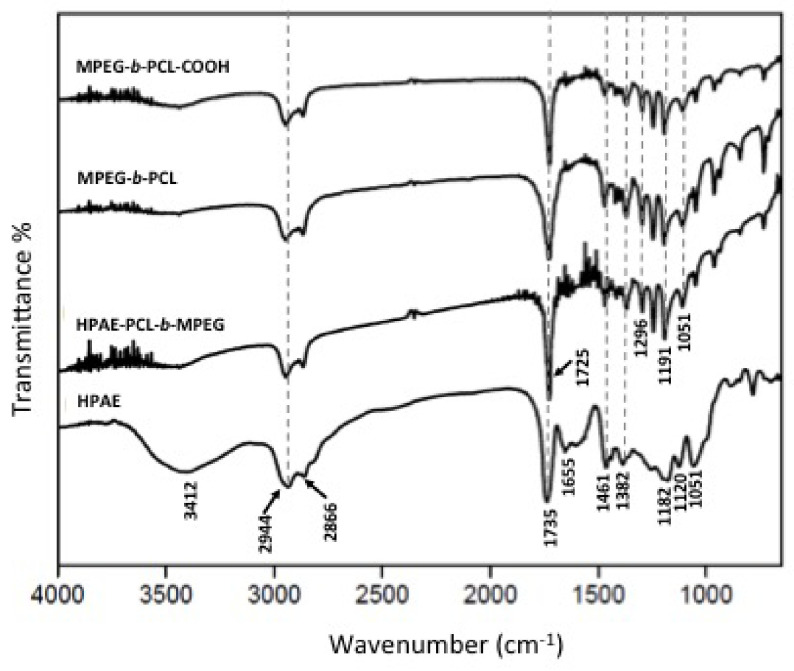
FTIR spectra of HPAE, MPEG-b -PCL, MPEG-b -PCL-COOH, and HPAE-PCL-b -MPEG.

In the FTIR spectra of HPAE (Figure 3), the broad peak around 3412 cm−1 suggests a large amount of terminal hydroxyl groups on the periphery of HPAE [20]. The absorption peaks at 1120 and 1051 cm−1 are assigned to C–N–C and C–O–C stretching vibrations, respectively. The peak at 1461 cm−1 corresponds to the asymmetric deformation vibration of the –CH2 – groups [21]. The peak at 1182 cm−1 corresponds to the C–O stretching vibration in HPAE [22]. Moreover, an ester carbonyl stretching band was observed at 1735 cm−1 . These results all demonstrated that the HPAE polymer was successfully synthesized.

**Figure 4:**
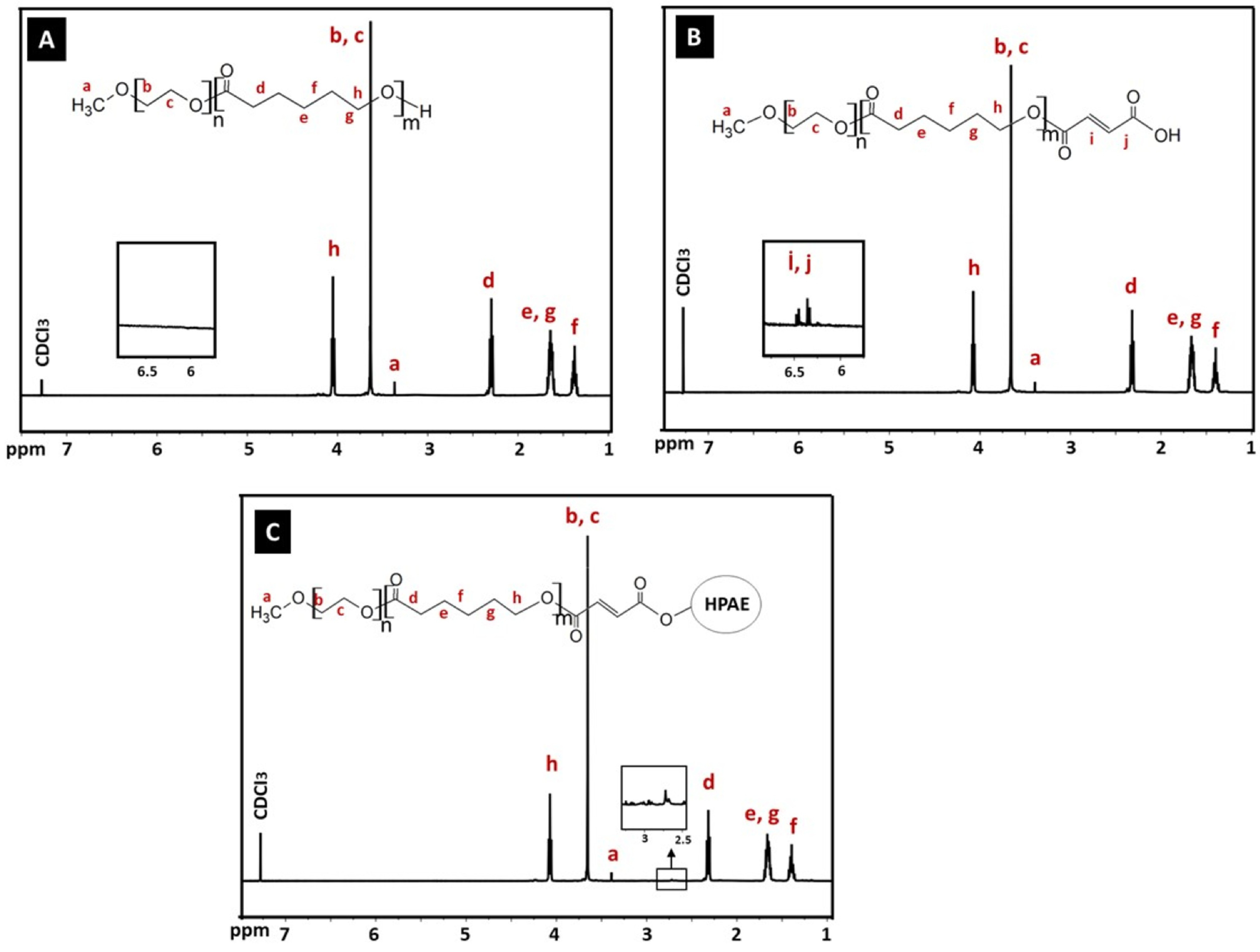
1 H NMR of (A) MPEG-b -PCL, (B) MPEG-b -PCL-COOH, and (C) HPAE-PCL-b -MPEG.

After the basic structure of HPAE was confirmed, the MPEG-b-PCL diblock polymer was synthesized by ring-opening polymerization of CL on MPEG chains in the presence of SnOct as a catalyst. To achieve high reactivity for further reactions, the next step was to convert hydroxyl groups of MPEG-b-PCL to carboxyl groups prepared by reacting MPEG-b-PCL with MA. The structures of MPEG-b-PCL and MPEG-b-PCL-COOH were identified by FTIR and 1 H NMR as given in Figures 3 and 4, respectively. The characteristic peaks of PEG and PCL on the MPEG-b-PCL diblock copolymer are shown at 1105 cm−1 and 1725 cm−1 due to the C-O stretching vibrations of the C-O-C bond and the C=O stretching vibrations of the ester group, respectively [21,23]. In addition, there are two peaks at 2944 cm−1 and 2866 cm−1 , which are due to the aliphatic C-H stretching band at the PCL and PEG blocks [24].

The HPAE-PCL-b-MPEG polymer was finally synthesized by reacting the carboxyl group of MPEG-b-PCL-COOH with the terminal hydroxyl groups of HPAE by an ester-forming reaction using EDAC and DMAP as the coupling agent and the catalyst, respectively. The formation of the HPAE-PCL-b-MPEG polymer was confirmed by FTIR and 1 H NMR (Figures 3 and 4). The stretching vibration of the terminal hydroxyl groups of HPAE was found at 3412 cm−1 , which significantly decreased in intensity of HPAE-PCL-b-MPEG with MPEGb-PCL substitution. In addition, the peak observed at 1182 cm−1 (C-O stretching vibration) in HPAE was slightly shifted to 1191 cm−1 as a stronger band in the MPEG-b-PCL, MPEG-b-PCL-COOH, and HPAE-PCLb-MPEG spectra. The sharp and strong peak at 1725 cm−1 was attributed to the C=O stretching vibrations of the ester group. These results indicate that the HPAE-PCL-b-MPEG was synthesized successfully.

The 1 H NMR spectrum of the synthesized MPEG-b-PCL copolymer is shown in Figure 4A. The characteristic peaks at 1.37, 1.64, 2.30, and 4.05 ppm were assigned to the methylene protons of –(CH2)3 –, –OCCH2 –, and –CH2 OOC– in the PCL blocks. Peaks at 3.37 and 3.63 ppm were attributed to the signals of CH3 – and –CH2 CHO– in the PEG blocks. The 1 H NMR spectrum of the synthesized MPEG-b-PCL indicated that the MPEG-b-PCL copolymer was successfully synthesized. The number average molecular weight (Mn) and PEG weight fraction of the synthesized MPEG-b-PCL block copolymer were calculated from 1 H NMR analysis according to the literature [25]. These were determined from the area ratio of the peak at 4.0 ppm (due to the PCL blocks), that of the peak at 3.58 ppm (due to the PEG block), and from the molecular weights of CL and EO repeat units, respectively [26]. The Mn of the synthesized MPEG-b-PCL copolymer was 6400 Da, which was very consistent with the theoretical Mn value of 7000 calculated according to the feed ratio of PEG/CL [27]. In addition, the molecular weight of MPEG-b-PCL, which was evaluated by GPC, was 7120 Da. As a result, the molecular weights detected from GPC and 1 H NMR could confirm each other. In addition, the intensity calculations obtained from NMR results confirm the formation of the PEG32 -PCL45 structure, which is also compatible with molecular weight results.

The 1 H NMR spectrum of the synthesized MPEG-b-PCL-COOH is shown in Figure 4B. The characteristic resonances of both PCL and PEG units were observed to be exactly the same as those of the spectrum of MPEG-b-PCL. In addition, two peaks at 6.44 and 6.56 ppm (-C(=O)CH=CHC(=O)-) are shown in the spectrum of MPEG-b-PCL-COOH, which are not found in the spectrum of the MPEG-b-PCL copolymer containing no MA units. Collectively, these results demonstrate the successful synthesis of the MPEG-b-PCL-COOH copolymer.

The 1 H NMR spectrum of the synthesized HPAE-PCL-b-MPEG is shown in Figure 4C. Compared with MPEG-b-PCL and MPEG-b-PCL-COOH, the 1 H NMR spectrum of HPAE-PCL-b-MPEG featured the appearance of a new peak at 2.73 ppm, referring to –CH2 COO– units of HPAE, which indicated the addition of HPAE to the MPEG-b-PCL-COOH copolymer. These results provide evidence that the newly synthesized HPAE-PCL-b-MPEG contains MPEG-b-PCL side chains.

### 3.2. Particle size and zeta potential analysis of HPAE-PCL-b-MPEG nanoparticles

**Figure 5:**
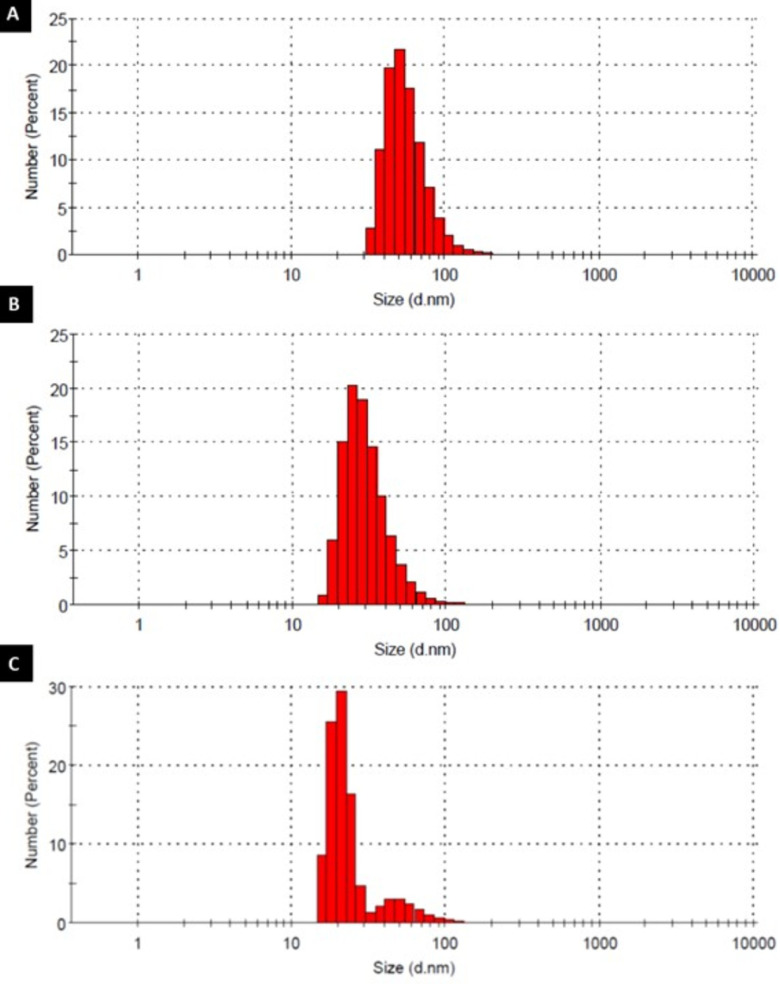
Particle size distribution of HPAE-PCL-b -MPEG nanoparticles prepared by different stirring rates: (A) 750 rpm, (B) 1000 rpm, and (C) 1200 rpm.

Highly branched, functionalized polymers have the potential to act as efficient drug nanocarrier systems. In this study, HPAE-PCL-b-MPEG nanoparticles were prepared by the nanoprecipitation technique [15] and their size was measured by dynamic light scattering (DLS). To optimize the size of HPAE-PCL-b-MPEG nanoparticles and determine the optimum process parameters, HPAE-PCL-b-MPEG nanoparticles were prepared under three different stirring rates (750 rpm, 100 rpm, and 1200 rpm). Small-sized nanoparticles were not obtained at a stirring rate below 750 rpm (data not shown). The stirring rate applied during the nanoprecipitation process affects the size of nanodroplets of the solvent formed during nanoprecipitation [28,29]. However, further increase in the stirring rate may cause an increase in particle size [28]. It can be clearly seen from Table 1 and Figure 5 that the size of HPAE-PCL-b-MPEG nanoparticles decreased gradually from ∼57.92 to ∼20.84 nm with the increase of the stirring rate from 750 to 1200 rpm. Two peaks are observed in Figure 5, which displays the occurrence of nanoparticle sizes for a stirring rate of 1200 rpm. According to the results, stirring rates between 750 and 1200 rpm had a slight influence on particle size, and 1000 rpm was selected as the optimum stirring rate due to the smaller size (30.97 ±12.51 nm) and polydispersity index (0.466) obtained. PDI is a measure of distribution of particle size. According to the literature, PDI greater than 0.5 indicates a polydisperse system, and if PDI is closer to zero, it indicates a monodisperse system [30–32]. In light of the literature, our results indicate that 0.466 is a favorable particle size (PDI < 0.5).

**Table 1:** Effect of stirring rate on the size of HPAE-PCL-b -MPEG nanoparticles.

Stirring rate (rpm)	Size (d. nm ±SD)	Polydispersity (PDI)
750	57.92 ±27.70	0.480
1000	30.97 ±12.51	0.466
1200	20.84 ±3.74 (peak 1) 55.64 ±27.20 (peak 2) 55.64±27.20 (peak 2)	0.474

In particular, particle size and surface behaviors have a significant role in passive targeting and the cellular uptake of nanoparticles. Torchilin et al. stated that nanoparticles of less than 200 nm are capable of spontaneous accumulations at the tumor region via enhanced permeability and retention effect and can be efficiently internalized through endocytosis by cancer cells [9,33,34]. As, within the scope of this study, the prepared HPAE-PCL-b-MPEG nanoparticles are smaller than 200 nm, we can say that HPAE-PCL-b-MPEG nanoparticles can be accumulated at tumor regions.

The measurement of zeta potential is critical in comprehending the surface charge characteristics of nanoparticles. A higher level of zeta potential results in greater electrostatic repulsion forces between the particles, which leads to larger separation distances between the particles, reducing aggregation formed by van der Waals bonding [35]. In this study, the zeta potential value of the HPAE-PCL-b-MPEG nanoparticles prepared under optimum conditions was measured by DLS and it was found to be +15.9 ±1.5 mV. It is well known that full electrostatic stabilization requires a zeta potential above 30 mV, while zeta potentials between 5 mV and 15 mV show limited stability and zeta potentials between –5 mV and +3 mV exhibit minimum stability [36]. As a result, the zeta potential value of HPAE-PCL-b-MPEG nanoparticles also suggests that the nanoparticles are homogeneously dispersed in the solution and do not aggregate much [37].

### 3.3. Characterization of 5Fu-loaded nanoparticles

**Figure 6:**
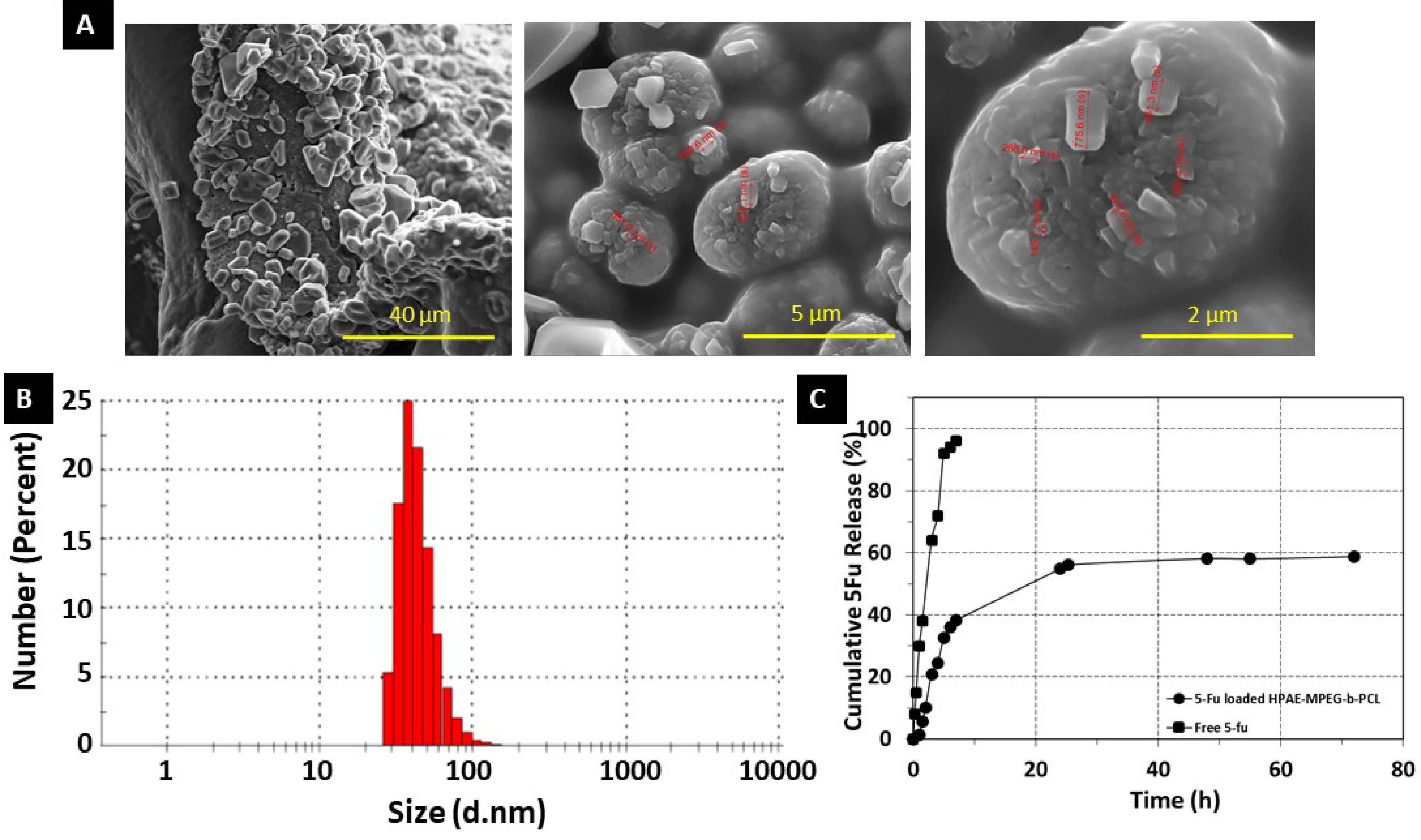
(A) SEM images of 5Fu-loaded HPAE-PCL-b -MPEG nanoparticles, (B) particle size of 5Fu-loaded HPAEPCL-b -MPEG nanoparticles in pH 7.4 PBS suspension, and (C) 5Fu release profile of HPAE-PCL-b -MPEG nanoparticles in PBS solution (pH 7.4) and free 5Fu diffusion across the dialysis tube for comparison.

SEM analyses were performed in order to characterize the morphology of 5Fu-loaded nanoparticles. Figure 6A shows the morphology of the freeze-dried 5Fu-loaded HPAE-PCL-b-MPEG nanoparticles. The surface of the dried nanoparticles is smooth, highly nonspherical, and hexagonally shaped. Unencapsulated 5Fu precipitate was not seen on the surface of the nanoparticles [38,39]. Therefore, it can be deduced that 5Fu was perfectly encapsulated in the HPAE-PCL-b-MPEG nanoparticles. The geometry of particles has a crucial effect on their blood circulation time [40]. Particularly, nanoparticles of nonspherical shape show long circulation times compared to spherical ones due to significantly reduced phagocytosis [41]. In addition, the size (d. nm) and zeta potential of 5Fu-loaded HPAE-PCL-b-MPEG nanoparticles were found to be about 44.81 ±16.73 nm (Figure 6B) and +11.3 ±0.8 mV. Nanoparticles can be accumulated at the tumor region via enhanced permeability and retention effect if their size is less than 200 nm [9,33,34] and their positively charged surface allows an electrostatic interaction between negatively charged cellular membranes and positively charged nanoparticles [42]. Consequently, hydrophilic shell, nonspherical shape, size, and zeta potential value of nanoparticles predict that the circulation time of HPAE-PCL-b-MPEG nanoparticles in the blood is partially extended. In addition, according to the literature, shapes with sharp features seem to be able to adhere to cancer cells better [43]. HPAE-PCL-b-MPEG nanoparticles have a hexagonal shape with sharp corners and edges, size less than 200 nm, and a positively charged surface, thus allowing them to easily deliver their cargo to cancer cells.

### 3.4. Drug loading and release studies

Drug loading content (DLC) and drug loading efficiency (DLE) of nanoparticles are two substantial elements to be appraised in terms of their in vitro or in vivo behaviors. Therefore, in this study, different formulations (polymer/drug weight ratio: 1/1, 1/0.5, 1/0.25, 1/0.1, and 1/0.05) were investigated to find the optimal DLC and DLE. The DLE was increased from 24.66% to 88.3% with increasing 5Fu concentration (Table 2). The optimal concentration of 5Fu for entrapment assays was found to be 63.24% in HPAE-PCL-b-MPEG nanoparticles. As the ratio of polymer/5Fu decreased, the DLC decreased from 548.05 to 81.83 mg/g, while the DLE gradually rose. The results reveal that 5Fu was easily encapsulated into the HPAE-PCL-b-MPEG nanoparticles with high DLC as well as satisfactory DLE due to interior-chemistry interaction, and 1/0.25 of the mass ratio of polymer/5Fu was chosen as an optimal formulation for further experiments.

**Table 2:** Drug loading content (DLC) and drug loading efficiency (DLE) of HPAE-PCL-b -MPEG nanoparticles.

Formulation	Polymer/drug weight ratio	DLC (mg/g)	EE (%)
F1	1/1	81.83	73.86
F2	1/0.5	195.20	88.3
F3	1/0.25	351.35	63.24
F4	1/0.1	539.04	48.51
F5	1/0.05	548.05	24.66

In vitro 5Fu release behaviors were evaluated in physiological condition (PBS, pH 7.4). As can be seen in Figure 6C, in vitro release of 5Fu from HPAE-PCL-b-MPEG nanoparticles is biphasic. It is worth noting that an initially rapid 5Fu release in the first 8 h (39% amount of drug) was followed by a controlled released in the second stage (20% amount of drug in 70 h). This is because of the decreasing concentration of 5Fu within the polymeric network after the initially rapid 5Fu release [44]. On the other hand, the characterization of in vitro drug burst release looks like a general phenomenon in numerous drug delivery platforms, as reported in the literature [45–52]. A similar biphasic release behavior has also been described for PLGA nanoparticles [53], chitosan microspheres [54], and poly(isobutyl-cyanoacrylate) nanoparticles [55]. In light of these similar findings, the obtained HPAE-PCL-b-MPEG nanoparticles are a promising candidate for drug delivery applications. On the other hand, subjected to the dialysis membrane method for assessing drug release, unencapsulated (free) 5Fu solution exhibited burst diffusion through the dialysis membrane, as seen in the peak-like profile displayed in Figure 6C [56]. These results confirm that HPAE-PCL-b-MPEG nanoparticles exhibit controlled release behavior.

### 3.5. In vitro cytotoxicity of HPAE-PCL-b-MPEG nanoparticles

**Figure 7:**
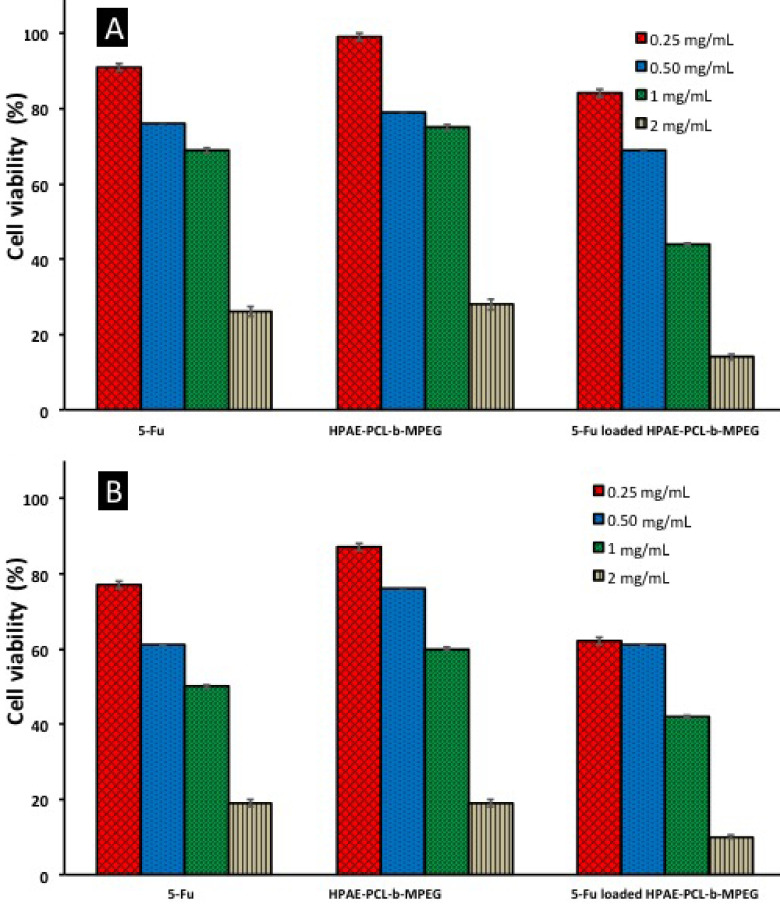
In vitro cytotoxicity of free 5Fu, blank, and 5Fu-loaded HPAE-PCL-b -MPEG nanoparticles against HCT116 cell lines using the MTT assay: (A) 24 h and (B) 48 h after treatment.

In vitro cytotoxicity of blank and 5Fu-loaded HPAE-PCL-b-MPEG nanoparticles was assessed by MTT assay. As can be seen in Figure 7, cell viability of blank and 5Fu-loaded HPAE-PCL-b-MPEG nanoparticles exhibited concentration-dependent and time-dependent effects against the HCT116 cell line. After 24 h and 48 h of treatment with HPAE-PCL-b-MPEG nanoparticles, HPAE-PCL-b-MPEG nanoparticles have less toxicity compared to 5Fu-loaded nanoparticles. Inhibition of cell growth is obvious at 1 mg/mL and 2 mg/mL after 24 h. The 5Fu-loaded HPAE-PCL-b-MPEG nanoparticles with concentrations of 1 mg/mL and 2 mg/mL have nearly 50% more inhibition than HPAE-PCL-b-MPEG nanoparticles. 5Fu-loaded HPAE-PCL-b-MPEG nanoparticles have less cell viability than blank nanoparticles. This indicates that blank HPAE-PCL-b-MPEG nanoparticles exhibit lower cytotoxicity than 5Fu-loaded nanoparticles, which was likely caused by 5Fu release. Moreover, 5Fu-loaded HPAE-PCL-b-MPEG nanoparticles exhibit better performance in achieving lower cell viability than the pure therapeutic drug 5Fu. As free 5Fu has a short half-life, the drug’s effect is rapidly lost in its administration [57].

The results of this study demonstrate that the IC50 value (50% inhibitory concentrations) for HPAE-PCLb- MPEG nanoparticles are about 1.48 ±0.05 mg/mL and 1.21 ±0.0531 mg/mL for 24 h and 48 h incubation periods, respectively (calculated from Figure 7). According to the literature, this value shows that HPAEPCL- b-MPEG nanoparticles are less toxic than PAMAM-G6 dendrimer (IC50 = 0.128 mg/mL) [58], PLGA nanoparticles (IC50 = 0.0329 mg/mL) [59], and gold nanoparticles (IC50 = 0.082 mg/mL) [60] on HCT116 cell lines.

## 4. Conclusions

In this study, a new amphiphilic core/shell-type hyperbranched polymeric nanocarrier platform (HPAE-PCL-b-MPEG) was designed as an effective drug nanocarrier system. The synthesized polymers were characterized with FTIR, 1 H NMR, 13 C NMR, and GPC analysis. HPAE-PCL-b-MPEG nanoparticles with average diameters of 30.97 ±12.51 nm were successfully prepared by the nanoprecipitation method. In addition, 5Fu-loaded HPAE-PCL-b-MPEG nanoparticles were successfully prepared by the same method, achieving superior drug loading content (351.35 mg/g), as well as satisfactory encapsulation efficiency (63.24%) due to interior-chemistry interaction. The particle size and zeta potential of 5Fu-loaded HPAE-PCL-b-MPEG nanoparticles were found to be about 44.81 nm ±16.73 and +11.3 ±0.8 mV. 5Fu-loaded HPAE-PCL-b-MPEG nanoparticles demonstrated a hexagonal structure and narrow size distribution. In addition, in vitro 5Fu release studies of HPAE-PCL-b-MPEG nanoparticles showed that there was an initially rapid drug release (39% of the drug in 8 h) followed by a controlled release in the second stage (20% of the drug in 70 h). Moreover, cytotoxicity results confirmed that the 5Fu-loaded HPAE-PCL-b-MPEG nanoparticles exhibit better performance in achieving lower cell viability than the pure 5Fu drug. These results demonstrate that HPAE-PCL-b-MPEG nanoparticles containing 5Fu can be a candidate for drug nanocarriers. In addition, we expect that conjugation of these nanoparticles with a targeting moiety could be a promising new drug carrier for actively targeted drug delivery systems in cancer therapy. Further studies on this area are currently underway in our laboratory.
